# Symptom provocation for treatment of obsessive-compulsive disorder using transcranial magnetic stimulation: A step-by-step guide for professional training

**DOI:** 10.3389/fpsyt.2022.924370

**Published:** 2022-08-03

**Authors:** Ana Maia, Sílvia Almeida, Gonçalo Cotovio, Daniel Rodrigues da Silva, Francisco Faro Viana, Jaime Grácio, Albino J. Oliveira-Maia

**Affiliations:** ^1^Champalimaud Research and Clinical Centre, Champalimaud Foundation, Lisbon, Portugal; ^2^Department of Psychiatry and Mental Health, Centro Hospitalar de Lisboa Ocidental, Lisbon, Portugal; ^3^NOVA Medical School, NMS, Universidade Nova de Lisboa, Lisbon, Portugal; ^4^Graduate Programme in Clinical and Health Psychology, Faculdade de Psicologia da Universidade de Lisboa, Lisbon, Portugal

**Keywords:** obsessive-compulsive disorder, transcranial magnetic stimulation, symptom provocation, structured training, guide

## Abstract

Transcranial Magnetic Stimulation (TMS) is a non-invasive brain stimulation technique that was cleared by the Food and Drug Administration (FDA) for the treatment of Obsessive-Compulsive Disorder (OCD) in 2018. The approved protocol includes individualized symptom provocation before each stimulation session, to elicit a moderate level of obsessional distress. Although symptom provocation can be a delicate, demanding, and uncomfortable procedure, structured training methods for those who are going to apply it are not available. Here, we describe a model for training in symptom provocation for TMS technicians, developed at the Champalimaud Clinical Centre in Lisbon, Portugal. Our programme includes two-sessions dedicated to clinical communication and symptom provocation techniques from a theoretical and practical perspective. Additionally, supervision meetings are conducted during treatment of patients, allowing regular case discussion and redefinition of symptom provocation hierarchy, as needed. In addition to having a strong practical component, our training program is short and pragmatic, allowing for easy implementation and fluid transition to clinical practice. By sharing our experience, we hope to contribute to systematize training procedures required for symptom provocation in the context of TMS, and to qualitatively describe a methodology that can be used for implementation of TMS programmes for the treatment of OCD.

## Introduction

Obsessive-Compulsive Disorder (OCD) is a severe psychiatric illness, phenomenologically characterized by the presence of obsessions and/or compulsions, that are time-consuming and cause significant functional impairment in personal, social and/or professional life (1). First-line approved treatments include cognitive-behavioral therapy (CBT) and/or pharmacological treatment such as selective serotonin reuptake inhibitors. Nonetheless, around 40% of patients are resistant to medication, 26% fail to initiate CBT, and 31% drop out before completing therapy (2, 3). Consequently, there is a need to develop new treatment strategies that are effective and tolerable. Transcranial magnetic stimulation (TMS) is a non-invasive brain stimulation technique that allows focal cortical neuromodulation in humans. It uses a rapidly changing magnetic field, created by electric current pulses flowing through a coil placed on the scalp, to induce an electric field in the target regions of brain. In 2018, TMS was cleared by the Food and Drug Administration for the treatment of OCD, using a protocol that includes individualized symptom provocation before each stimulation session, to elicit a moderate level of obsessional distress, as reported by patients (4).

Despite the absence of controlled trials for symptom provocation in TMS treatment of OCD, evidence from studies using TMS for smoking cessation (5), depression (6) and post-traumatic stress disorder (7) suggest that symptom provocation significantly influences clinical outcomes. In theory, symptom provocation induces the reconsolidation of fear and distressing memories into long-term memories, which can be disrupted by neural stimulation during this susceptible period (8–11). Provoking obsessive-compulsive symptoms is associated with activation of the cortico-striato-thalamo-cortical circuitry, mainly in the right hemisphere (12, 13), which can be selectively modulated by TMS. Indeed, evidence suggests that the efficacy of brain stimulation is dependent not only on the characteristics of stimulation but also on the initial state of neural activation in the target brain region (14). Importantly, targeting circuits that are functionally activated by symptoms is thought to increase the specificity of TMS-induced plasticity, since it may allow for preferential modulation of the neural populations that are most affected by symptoms and distress (14, 15). However, this procedure is delicate, clinically demanding, and usually uncomfortable for both the patient and the TMS technician. As the use of TMS for the treatment of neuropsychiatric disorders continues to grow, there is a need to share and systematize methodologies on such therapeutic strategy. While there is literature describing symptom provocation administration in the context of TMS (16), orientations on structured training methods in symptom provocation for TMS technicians are still missing.

In our clinical center, a team of physicians and psychologists with specialized training in behavioral medicine and CBT developed a two-session programme to train TMS technicians in the administration of the symptom provocation procedure. In this step-by-step guide, we intend to describe this method in detail, from a theoretical and practical perspective.

## Materials and Methods

### A two-session model for a pragmatic training method

**Figure 1:**
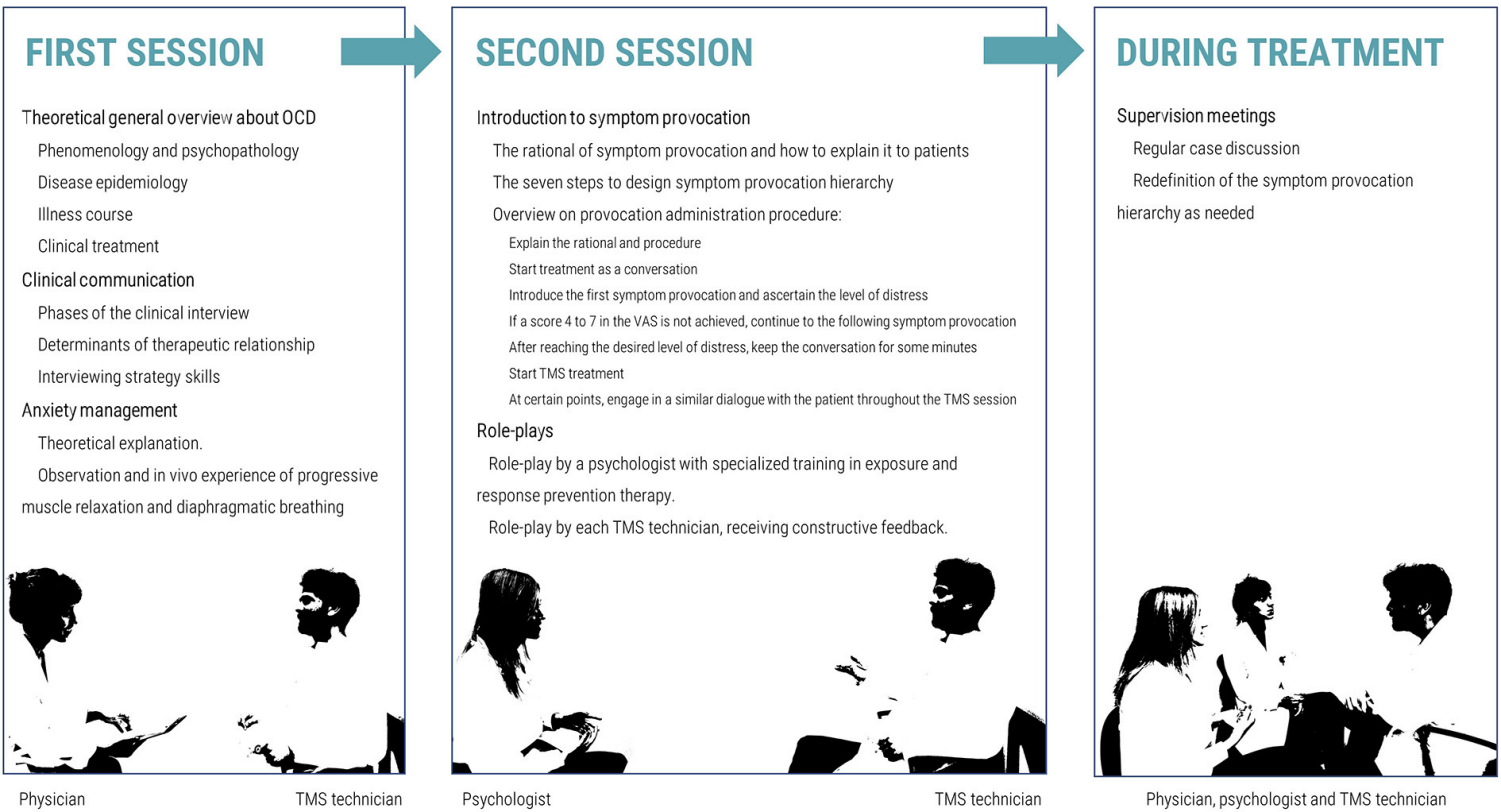
Diagram illustrating the two-session model for training in symptom provocation.

We designed a training method including two sessions of approximately 3 h each, conducted 1 week apart (Figure 1). This brief and pragmatic methodology was designed to ensure that TMS technicians are provided with specialized theoretical and practical knowledge, not only regarding symptom provocation procedure itself, but also regarding the nature of OCD, clinical communication techniques and anxiety management techniques. Before each session, theoretical material (16–18) was shared with TMS technicians, who were instructed to study this material beforehand. The use, distribution and reproduction of those materials are protected by copyright and should only be used in accordance with the terms provided by the original author(s) and the copyright owner(s).

### First session

#### Overview of obsessive-compulsive disorder

The first session started with a theoretical general overview about OCD, provided by a clinician experienced in managing patients with this condition. A detailed description of phenomenology and psychopathology of obsessions and compulsions, disease epidemiology, illness course, and clinical treatment, according to the most recent guidelines, was presented (1, 19). A particular emphasis was placed on the phenomenology of OCD, as it is crucial to properly understand the nature of obsessive-compulsive symptoms to provoke obsessions, but also to identify compulsions, which should be avoided during the symptom provocation procedure.

#### Clinical communication techniques

Subsequently, a clinical psychologist with specialized training in behavioral medicine and CBT conducted a brief training in clinical communication skills. After an overview about the phases of the clinical interview and the determinants of therapeutic relationship, specific interviewing strategy skills were explored, such as active listening, empathic responses, validation, reassurance, summarization, guided-questioning techniques and non-verbal communication (17). Lastly, anxiety management techniques were explored at a theoretical level, and modeled through observation and *in vivo* experience, namely progressive muscle relaxation and diaphragmatic breathing (20).

### Second session

#### Introduction to symptom provocation

The second training session, again with the clinical psychologist, began with an introduction to the rational of symptom provocation, as well as its procedures. Provoking obsessive symptoms deliberately might be uncomfortable for both the patient and technician. Therefore, it is extremely important to clarify the procedure in detail with the technicians, including a mention to the mechanistic rationale that is thought to underlie the benefits of symptom provocation. This point is also important because it should be discussed with patients prior to applying symptom provocation. It is crucial to explain to the patient that, although distressing in short term, the purpose of symptom provocation is to improve long-term treatment effectiveness. This information should be recalled by the TMS technicians across treatment cycle as needed. For instance, technicians might mention to patients: “*By provoking obsessive symptoms, certain areas and circuits of your brain will be activated, which should make them more susceptible to stimulation. Despite being unpleasant in the short term, this procedure aims to enhance clinical improvement”*.

**Figure 2:**
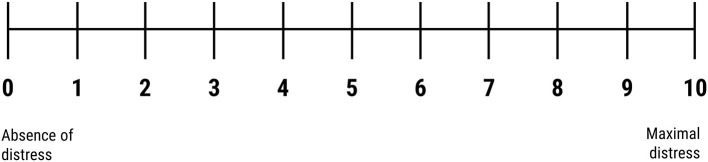
Visual Analog Scale.

Technicians were familiarized with all procedures necessary for symptom provocation, including the process of designing a symptom provocation hierarchy, which is performed prior to the TMS by a trained clinician. Although technicians are not directly involved in this step, they should be acquainted with the seven-steps process of provocation hierarchy design, namely (1) applying the Yale-Brown Obsessive Compulsive Scale (YBOCS) symptom checklist to identify primary symptoms, (2) creating a draft list of the primary symptoms, (3) completing the YBOCS severity scale, (4) creating a symptom hierarchy from the draft list, developing (5) internal and (6) external provocations from the least to the most distressing obsessions in symptom hierarchy, and (7) reviewing with the technician the nature of the patient's specific obsessive-compulsive symptoms, and the individualized symptom provocation hierarchy. This training session also included an overview on provocation administration procedure, namely how the technicians should use the symptom provocation hierarchy to intentionally elicit a moderate self-reported level of obsessional distress (i.e., “4 to 7” in a “0 to 10” Visual Analog Scale, as illustrated in Figure 2) before each stimulation session. Despite the inexistence of controlled studies assessing the ideal level of subjective self-reported distress, this Visual Analog Scale interval was defined according to clinical experience and was associated with a good acceptability and treatment efficacy in clinical trials that lead to clearance of TMS for the treatment of OCD (21). As described by Tendler and colleagues (16), symptom provocation should be administered using internal and external hierarchy provocation list as a guide to formulate questions that instills obsessive distress until the desired level of self-reported distress is achieved. TMS technicians should start from the first provocation in the list, which is the least distressing, and move progressively to more distressing provocations. If needed, technicians can combine internal and external provocations to achieve the desired level of obsessional distress.

It is particularly important to underline practical recommendations when dealing with difficult situations. For instance, if the patient is excessively distressed with a distress score higher than 7 in the Visual Analog Scale, technicians should withhold TMS until it is possible to reduce the level of distress to the desired levels. This can be achieved by talking about a different topic or simply by remaining silent while allowing the patient to recover. If necessary, the technician and/or the patient can leave the room momentarily, although it is important to remind the patient that he/she should not complete any anxiety reducing compulsion. In the second session, the symptom provocation procedure should be explored according to previously published guidelines (16) and should ideally include the contribution and feedback of technicians with experience in obsessive symptom provocation.

#### Role-plays

Lastly, role-plays illustrating the symptom provocation procedure were performed using clinical case vignettes specifically designed for the course. Evidence suggests that role-plays in supervised groups increase therapeutic and communication skills, not only for participants assuming the roles but also for the observers (22). A first role-play was performed by a psychologist with specialized training in exposure and response prevention therapy. Finally, all TMS technicians had the opportunity to perform role-play and receive constructive feedback. For illustrative purposes, we will exemplify two role-plays using two fictitious clinical vignettes.

### Case 1

*Patient M. is a 38-year-old woman who works as a designer and lives alone. Symptoms started at 6 years-old, mainly with mild symmetry obsessions, and ordering compulsions. At the beginning of her adulthood, she developed contamination and somatic obsessions with cleaning, washing and checking compulsions, mainly characterized by excessive fear of being infected with Human Immunodeficiency Virus (HIV) and compulsive handwashing and checking of the skin for Kaposi's sarcoma lesions. Symptoms caused a gradually increasing impairment in professional and social life, leading to search for psychiatric help at the age of 30. During the last 8 years, the patient was treated with sertraline 100 mg/day for 3 years, sertraline 200 mg/day for 2 years, paroxetine 60 mg/day for 1 year, paroxetine 60 mg/day plus aripiprazole 15 mg/day for 1 year, and CBT for 3 years. Although there were periods of partial remission, symptoms persisted and a severe relapse in the last year impaired her ability to work and seriously compromised her social life. Consequently, medication was augmented to paroxetine 60 mg/day plus aripiprazole 30 mg/day, but only a mild response was observed after 6 months of psychopharmacology and co-adjuvant CBT. Consequently, an acute cycle of TMS was proposed, including 30 daily sessions of high-frequency TMS over the dorsomedial prefrontal cortex*.

*Before the first session, patient met a clinical psychologist to complete the seven-step process of provocation design. The psychologist explained in detail the rationale for symptom provocation and procedure, explaining that the aim of the appointment was to jointly define a list of symptom provocation that would be used by the TMS technician in the beginning of each TMS session. The psychologist specifically mentioned that she would meet with the TMS technician to discuss the case and the symptom provocation list. Using the YBOCS severity scale, the following hierarchy of symptom provocation was designed (from the least to the most distressing):*

*1. “When was the last time you were in contact with someone that you thought could be infected with HIV?” – Internal provocation*.

*2. Show the word “HIV” written in a paper and ask: “how much does it bother you when you look to this word?” – External provocation*.

*3. “Did you examine your skin this morning? did your check if you have any lesion? are you sure?” – Internal provocation*.

*4. “How did you come here? do you think you may have contracted HIV on the way?” – Internal provocation*.

*5. “Did you wash your hands when you got here? are you sure you washed them properly?” – Internal provocation*.

*6. “When was the last time you did an HIV test? are you sure everything was ok? did you check the clinical report?” – Internal provocation*.

*7. Show the picture of a kaposi sarcoma and say: “this is a kaposi sarcoma. Is it possible that you had an identical lesion that you did not detect so far?” - External provocation*.

*After meeting with the patient, the psychologist presented the case to the TMS technician, including a complete personal history and a detailed overview of the symptom provocation hierarchy*.

After reading the clinical vignette, TMS technician starts the role-play by simulating symptom provocation procedure during the first TMS session. A successful role-play must include several specific steps, as exemplified in Table 1.

**Table 1:** Role-play case 1.

**1. Explaining the rationale:**
TMS technician:*“Symptom provocation is the intentional induction of a moderate level of self-reported obsessional distress before TMS treatment. By provoking obsessive symptoms, certain areas of your brain will be activated, which will make them more susceptible to stimulation. Despite unpleasant in the short term, this procedure aims to enhance clinical improvement”*.
**2. Explaining the procedure:**
TMS technician: “*During this procedure, we will talk about your symptoms, which were presented to me by the clinical psychologist that met with you some days ago. As I just mentioned, for this treatment it is necessary to elicit in you a certain level of distress, which shouldn't be too extreme, nor too subtle. Consequently, I will be asking you regularly, on a scale from 0 to 10, on how distressed you feel, where 0 corresponds to the absence of distress and 10 to the maximum distress that you can imagine. Higher or lower levels of distress are not necessarily better, so it is important that your answers are as precise as possible. This level of distress must be maintained during the session, which will last around 20 minutes. Symptom provocations will be organized from the least to the most distressing, and it is important that you do not perform any compulsions or behaviors to reduce distress until the end of the session. Throughout the treatment cycle, I will keep in touch with the psychologist that has interviewed you and I will be here to help you in all sessions. Do you have any questions?*” Patient M.: “*No, I don't*.”
**3. Start the treatment as a conversation to engage and find information that can be used to provoke symptoms:**
TMS technician: “*Before starting, I would like to know you better. Can you briefly present yourself?*” Patient M.: “*Yes, I'm 38-years-old. I live alone and I work as a designer. However, I stopped working a few months ago because I cannot go outside without thinking that I will get HIV if I touch anything that was touched by someone infected*.” TMS technician*:* “*So you are avoiding leaving home. Can you briefly describe a typical day for you?*” Patient M.: “*Before, I used to go to the office and meet some friends after work. However, in the last weeks I've been essentially in my apartment completely alone*.”
**4. Explore obsessive symptoms:**
TMS technician: “*Thinking constantly that you might be infected with HIV when you go out must be extremely distressing. Can you tell me more about that?*” Patient M.: “*I started thinking about this in my early 20's, after a colleague in university had been diagnosed with HIV. Since then, this fear never disappeared. Basically, I'm afraid of getting in contact with someone that has HIV or touching a surface that could have been contaminated.”*
**5. When appropriate, introduce the first symptom provocation and ascertain the level of distress:**
TMS technician: “*When was the last time you were in contact with someone you though that could have HIV?*” (Internal provocation) Patient M.: “*Yesterday I went to the supermarket and I was afraid that the lady in front of me had HIV. She didn't look good, and she disinfected her hands too many times*.” TMS technician: “*I see. From a scale from 0 to 10, how much distress do you feel when thinking about that situation?*” Patient M.: “*Not much, yesterday I felt quite anxious but today not that much, maybe a 2*”.
**6. Since a score between 4 and 7 in the Visual Analog Scale was not achieved, continue to the following symptom provocation according to the symptom provocation hierarchy:**
TMS technician: “*Now I would like to ask if you can look at this word*.” Show the word ‘HIV' written in a paper. “*From a scale from 0 to 10, how much does it bother you looking to this word?*” (External provocation) Patient M.: “*I would say a 3*”. TMS technician: “*The psychologist also told me that when you are out you check all surfaces where you touch. Did you examine every surface you touched today?*” (Internal provocation) Patient M.: “*Yes, I checked the seat of the taxi*”. TMS technician: “*Did you check it properly? Are you sure that there wasn't any blood spot in there?*” Patient M.: “*Well… I can't be absolutely sure…*”. TMS technician: “*From a scale from 0 to 10, how much distress do you feel when thinking about this?*” Patient M.: “*It's very distressing… probably a 5 or 6*”
**7. After reaching the desired level of distress (score between 4 and 7 in the Visual Analog Scale), the TMS technician should keep the conversation for some minutes to prevent patients from understanding which the required distress level is, to allow for the beginning of the treatment. Start TMS treatment:**
TMS technician: “*Ok, now if you're ready, I will start the treatment. Please keep in mind not to perform any compulsion or other behaviors to reduce distress until the end of the session*.”
**8. At certain points throughout the TMS session, the technician should engage in a similar dialogue with the patient to assess the current intensity of distress and, if needed, adjust it by using the techniques already mentioned**.

### Case 2

*Patient L. is a 25-year-old man who lives with his parents and is unemployed. His first obsessive symptoms started in the beginning of adolescence and were characterized by scrupulosity and aggressive obsessions such as fear of offending or hurting others by acting on unwanted impulses. Consequently, he developed mental compulsions characterized by ritualized thoughts aiming to cancel the obsessive thoughts, such as repeating the following sentence in his thoughts, an even number of times: “I will not harm anyone, I am not a bad person, I am a good person”. At 22 years-old, symptom intensity and frequency increased, and patient started to avoid leaving his house. He abandoned university and became severely isolated. At this point, he started being followed by a psychiatrist and, at 23 years-old was diagnosed with OCD and started treatment with CBT and escitalopram, titrated up to 40 mg/day. However, only a mild response was observed, and the patient was then treated with aripiprazole 10 mg/day and then risperidone 3 mg/day, both discontinued due to side effects. Consequently, an acute cycle of 30 daily high-frequency TMS sessions over the dorsomedial prefrontal cortex was proposed. In the psychology consultation before the first session, the rational and procedure of symptom provocation were carefully explained. After explaining that the aim of that consultation was to define together a list of provocations that would be used by the TMS technician to provoke obsessive symptoms, the psychologist applied the YBOCS severity scale and designed the following hierarchy of symptom provocation (from the least to the most distressing):*

1. “*When you feel afraid of offending someone, do you think there is a risk of actually doing it?” – Internal provocation*
2. “*If you would be aggressive with someone, what would you do?” – Internal provocation*
3. “*Now I will ask you to think about hurting someone on your way home without a motive” – Internal provocation*
4. “*Can you start one of your mental compulsions to reduce the anxiety that you are experiencing, but stop it on an odd number of repetitions? How does that make you feel?” – External provocation*
5. “*As you have all these aggressive thoughts in your head, do you think you can actually be a bad person?” – Internal provocation*
6. *Show the sentence “I am a bad person” written on a paper. “Now I would like you to look to this sentence. How do you feel?” - External provocation*.
7. *Keep showing the sentence “I am a bad person” written on a paper. “Can you read this sentence out loud? How do you feel?” - External provocation*.

After reading the clinical vignette, TMS technician starts the role-play by simulating symptom provocation procedure during the first TMS session. As mentioned in Case 1, a successful role-play should cover several specific topics, as exemplified in Table 2.

**Table 2:** Role-play case 2.

A successful role-play should start by explaining the rationale and procedure of symptom provocation (points 1 and 2 described above for Case 1). Next, the following topics should be covered:
**3. Start the treatment as a conversation to engage and find information that can be used to provoke symptoms:**
TMS technician: “*Before starting, I would like to know you better. Can you briefly present yourself?*” Patient L.: “*Sure, I am 25 years-old and I live nearby with my parents and my younger brother. I started studying Agronomy, but I had to quit university due to my symptoms*.” TMS technician: “*How did your disease make you quit university?* Patient L.: ”*I started being really afraid of offending or hurting someone when I was around other people, and these thoughts were particularly intense when I was around people outside my family, particularly people that I admired and respected, like some teachers or close friends. Consequently, I started avoiding leaving my place and eventually I was not able to go out to the university*.”
**4. Explore obsessive symptoms:**
TMS technician: “*Can you tell me more about those thoughts?*” Patient L.: “*I just can't avoid thinking that I will offend or hurt these people in a violent way. I don't think I want to do it, but I just can't avoid thinking about it*.”
**5. When appropriate, introduce the first symptom provocation and ascertain the level of distress:**
TMS technician: “*When you feel afraid of offending someone, do you think there is a risk of actually doing it?*” (Internal provocation) Patient L.: “*I don't know. On one hand I have these thoughts all the time and I have never offended any of these people. On the other, I just can't avoid thinking about it so I would say that the risk can't be zero*.” TMS technician: “*From a scale from 0 to 10, how much distress do you feel when thinking about that possibility?*” Patient L.: “*Maybe a 3. As my psychologist told me, thinking something is not the same as doing it*.”
**6. Since a score between 4 and 7 in the Visual Analog Scale was not achieved, continue to the following symptom provocation according to the symptom provocation hierarchy:**
TMS technician: “*You told me that you were also afraid of hurting someone. Can you tell me more about these thoughts?*” Patient L.: “*Yes, I keep thinking that I can be really aggressive to some of these people that I admire. Often, I even have images coming to my mind of violent aggressions in which I am hurting these people very badly*.” TMS technician: “*Can you describe these images in detail?*” (Internal provocation) Patient L.: “*I frequently think of strangling them with my own hands. It's horrible, I know*.” TMS technician: “*That must be very distressing for you. From a scale from 0 to 10, how distressing are these thoughts?*” Patient L.: “*A 5, these thoughts and images are just awful*.”
**7. After reaching the desired level of distress (score between 4 and 7 in the Visual Analog Scale), the TMS technician should keep the conversation for some minutes to prevent patients from understanding which the required distress level is, to allow for the beginning of the treatment. Start TMS treatment:**
TMS technician: “*Ok, now if you're ready, I will start the treatment. Please keep in mind not to perform any compulsion or other behaviors to reduce distress until the end of the session*.”
**8. At certain points throughout the TMS session, the technician should engage in a similar dialogue with the patient to assess the current intensity of distress and, if needed, adjust it by using the techniques already mentioned**.

Role-plays must run without interruptions. After each role-play the psychologist administrating the training invites all group members to give their opinions about the performance with constructive feedback.

#### Supervision meetings

During TMS treatment cycles, supervision meetings with a physician and a psychologist were performed, allowing regular case discussion and redefinition of symptom provocation hierarchy, if needed. As the patient improves across treatment, provocations that were initially effective in eliciting a moderate self-reported level of obsessional distress, may become insufficient. In this case, the psychologist that designed symptom provocation hierarchy should redefine this list by including new symptom provocations, as appropriate according to clinical improvement, using clinical information collected during the first assessment. For instance, in the second role-play described above (Case 2), in the first TMS session the TMS technician achieved a score between 4 and 7 in the Visual Analog Scale using the second provocation in the hierarchy. However, if, as the patient improves, the 7 provocations in the symptom provocation hierarchy become insufficient to elicit the desired level of distress, the addition of more distressing provocations by the psychologist would be needed. It should be noted that symptom provocation hierarchies are usually more extensive (around 20 to 30 items, in our clinical center) and that the provocation hierarchies in the two cases described here were fictitiously designed merely for illustrative purposes.

## Results and discussion

Obsessive-compulsive symptom provocation is a challenging clinical procedure and an important part of FDA-cleared procedures for TMS treatment of OCD (4, 21). Despite the growing use of symptom provocation, additional research is necessary to confirm its benefits for TMS treatment of OCD, and to refine the procedure's methodology. Indeed, to our knowledge, there are currently no controlled studies comparing TMS for OCD with and without symptom provocation or using different symptom provocation methods. Nevertheless, experimental paradigms in animal models and human studies theoretically support symptom provocation effectiveness (8–14, 23, 24), and clinical studies that assessed TMS for OCD using symptom provocation, on which FDA clearance was based, achieved favorable outcomes with a good acceptability (21, 25). However, we are unaware of any available guidelines on training methods in symptom provocation for TMS technicians. Additionally, to date, no study has assessed the influence of individualization of symptom provocation on the effectiveness of TMS treatment. Indeed, obsessive-compulsive symptoms are highly heterogenous in terms of frequency, intensity and thematic content (26), and psychopathological variability has an impact on symptom induction procedures. Putter and colleagues performed a meta-analysis on the efficacy of provocation procedures in OCD and found larger effect sizes for threat and responsibility provoking methods, and when stimuli were tailored to individuals (27), supporting the use of an individualized list of symptom provocation rather than a standard script for all patients. Furthermore, given that different obsessive-compulsive dimensions are thought to be mediated by distinct components within the cortico-striato-thalamo-cortical circuitry (28), it is reasonable to hypothesize that the activation of specific brain circuits involved in individualized symptom may increase the specificity of TMS-induced plasticity. This can be particularly beneficial when using coils that induce a less focal brain modulation, such as the H7 coil approved by the FDA for TMS treatment of OCD (21, 25, 29, 30). However, designing and administering individualized symptom provocation is highly time-consuming and requires a more subtle, oriented, and flexible symptom provocation by TMS-technicians. Lastly, differences in the theoretical knowledge of different TMS technicians and the subjectivity involved in these processes might also influence the effectiveness of the symptom provocation procedure. Nevertheless, individualized symptom provocation is a part of the FDA-cleared TMS protocol for the treatment of OCD (4), and it is expected to be increasingly used given the rapid growth of these more interventional therapeutic procedures in psychiatry. Thus, systematized training methods in symptom provocation procedure are needed, in order to maximize safety and effectiveness, while minimizing variability between TMS technicians and centers.

We developed a two-session methodology for training in obsessive symptom provocation for TMS technicians, addressing clinical communication and symptom provocation techniques from a theoretical and practical perspective. In our clinical center, all TMS technicians have been trained using this training methodology before administering TMS to patients with OCD. To date, all patients experienced a good acceptability of symptom provocation procedure and a good progression in symptom provocation hierarchy across sessions, which represents an indirect measure of treatment efficacy. For instance, among three initial patients completing the TMS protocol for OCD, the average and maximal number of the provocation in the hierarchy list needed to induce a 4 to 7 self-reported score in the Visual Analog Scale was consistently higher in the second half of the acute treatment, as demonstrated in Table 3.

**Table 3:** Progression in symptom provocation hierarchy across session of three patients with OCD treated with TMS in our clinical center.

		**Patient 1**	**Patient 2**	**Patient 3**
**Sex, age**		Male, 36	Male, 30	Female, 49
Number of items in symptom hierarchy		13	23	11
Sessions 1 to 14	Mean	3.3	12.5	5.6
	Maximum	5	17	7
Sessions 15 to 30	Mean	8.0	15.9	9.9
	Maximum	8	18	11

As an example, patient 1 was a 36 years-old man with obsessive thoughts mainly focused on right and wrong moral content, scrupulosity, blasphemy, and sacrilege. Symptom provocation hierarchy had a total of 13 items. While in the first half of treatment, the average number of symptom provocation used was number 3 (which corresponds to asking the patient “*When was the last time that you said something wrong to someone? Are you sure you didn't hurt that person?*” – Internal provocation), in the second half, the average number of symptom provocation used was provocation number 8 (showing the word Sinner written in a paper and ask “*How much does it bother you to look at this word?”* – External provocation).

Our training program has a strong practical component, allowing for a more fluid transition to clinical practice. Furthermore, the program is short and pragmatic, and it does not require previous experience in dealing with patients with OCD, allowing an easy implementation by trained physicians and psychologists, independently of their background. By sharing this work, we hope to contribute to systematize training procedures required for TMS administration, and to qualitatively describe a methodology that can be used as a model for other centers when implementing TMS clinical programs for OCD.

## Data Availability Statement

The original contributions presented in the study are included in the article, further inquiries can be directed to the corresponding author.

## Ethics Statement

Written informed consent was obtained from the individual(s) for the publication of any potentially identifiable images or data included in this article.

## Author contributions

AM, SA, GC, DR, FV, JG, and AO-M formulated the training protocol in symptom provocation for TMS technician. AM wrote the manuscript, that was critically reviewed, and approved by SA, GC, DR, FV, JG, and AO-M. AO-M supervised project planning and execution. All authors contributed to the article and approved the submitted version.

## Funding

AM and GC were supported by doctoral fellowships from Fundação para a Ciência e Tecnologia (FCT, references SFRH/BD/144508/2019 and SFRH/BD/130210/2017, respectively). GC and AO-M were supported by grant PTDC/MED-NEU/31331/2017 from FCT and AO-M was supported by grant FCT-PTDC/MEC-PSQ/30302/2017-IC&DT-LISBOA-01-0145-FEDER, funded by national funds from FCT/Ministério da Ciência, Tecnologia e do Ensino Superior (MCTES) and cofounded by Fundo Europeu de Desenvolvimento Regional (FEDER), under the Partnership Agreement Lisboa 2020—Programa Operacional Regional de Lisboa. This work was supported by the Brain and Behavior Research Foundation (BBRF) through grant BBRF-27595-2018 NARSAD to AM and AO-M. FCT/MCTES, FEDER, and the BBRF did not have a role in the design and conduct of this work, in the preparation, review, or approval of the manuscript, nor in the decision to submit the manuscript for publication.

## Conflict of interest

Author AO-M was national coordinator for Portugal of a non-interventional study (EDMS-ERI-143085581, 4.0) to characterize a Treatment-Resistant Depression Cohort in Europe, sponsored by Janssen-Cilag, Ltd. (2019-2020), is recipient of a grant from Schuhfried GmBH for norming and validation of cognitive tests, and is national coordinator for Portugal of trials of psilocybin therapy for treatment-resistant depression, sponsored by Compass Pathways, Ltd. (EudraCT numbers: 2017-003288-36 and 2020-001348-25), and of esketamine for treatment-resistant depression, sponsored by Janssen-Cilag, Ltd. (EudraCT number: 2019-002992-33). The remaining authors declare that the research was conducted in the absence of any commercial or financial relationships that could be construed as a potential conflict of interest.

## Publisher's note

All claims expressed in this article are solely those of the authors and do not necessarily represent those of their affiliated organizations, or those of the publisher, the editors and the reviewers. Any product that may be evaluated in this article, or claim that may be made by its manufacturer, is not guaranteed or endorsed by the publisher.
